# Colonoscopic retrieval of migrated copper-T

**DOI:** 10.4103/0972-9941.55107

**Published:** 2009

**Authors:** Laleng M Darlong, Subrat Panda, Noor Topno, Ranendra Hajong

**Affiliations:** Department of Surgery, NEIGRIHMS, Shillong, India; 1 Department of Obstetrics & Gynecology, NEIGRIHMS, Shillong, India

**Keywords:** Colonoscopy, migrated Cu-T

## Abstract

Intrauterine contraceptive devices have been in use for a long time as family planning measures, one of its complications of perforating the uterus and migrating into the peritoneal cavity is also well known. Retrieval in such cases depends on the location of the migrated intrauterine devices and involves laparotomy or laparoscopy. We present here such a case that migrated partially into the lumen of the rectosigmoid and was successfully removed using a colonoscope.

## INTRODUCTION

Intrauterine contraceptive device (IUCD) has been in vogue since 1965^1^ for the purpose of contraception. Perforation of the uterus is not infrequent and occurs in 1/350–1/2500 insertions. Perforation leads to migration of the device to the organs to which the uterus is intimately related, such as the bladder and the rectosigmoid. Perforation may occur during the act of insertion or at a later date. Perforation during insertion is directly related to the experience and skill of the clinician. We report a case of IUCD which had perforated partly into the rectosigmoid and partly lying in the pelvic cavity, which was successfully removed using a colonoscope.

## CASE REPORT

A 25-year-old women; Para 2 and abortion 1 presented to the Obstetrics and Gynecology OPD with a history of a copper T insertion in January 2004. Since December 2004, she could not feel the thread. She had a history of missed period and consulted a local gynecologist. Pregnancy was confirmed and a MTP was done for the unwanted pregnancy. During the MTP, IUCD was not found in the uterine cavity. At this point, the patient was referred to our center for further management.

She complained of constant backache and pain during defecation.

**Figure 1:**
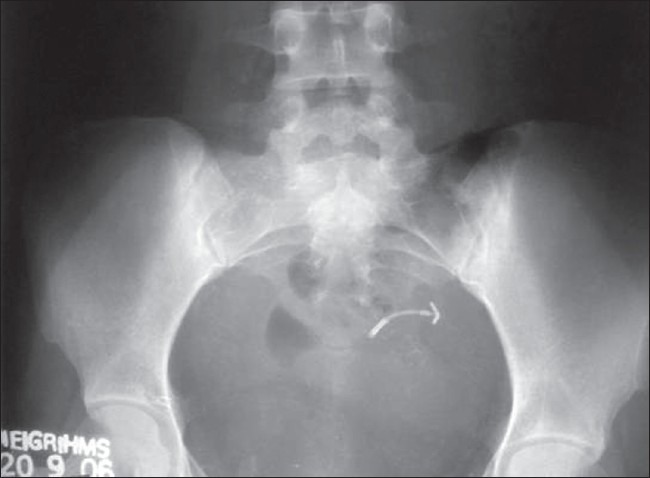
Migrated Cu-T in plain X-ray pelvis

**Figure 2:**
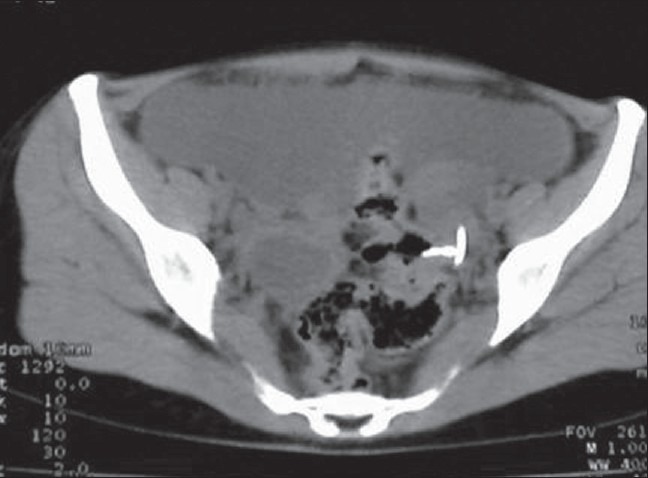
CT scan showing IUCD in the rectum

**Figure 3:**
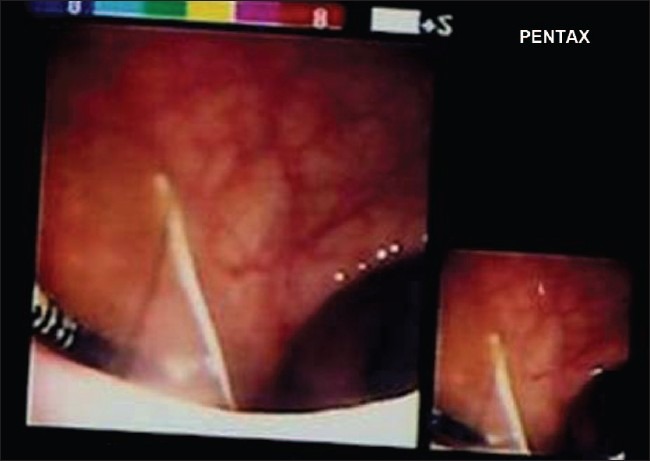
One of the T limb of Cu-T in rectum

Clinical examination revealed tenderness in the lower abdomen on deep palpation, no rebound tenderness, no free fluid; P/V: tenderness in the posterior fornix. P/R: mild tenderness was elicited. Pelvic X-ray showed the IUCD lying outside the uterus [Figure 1]. USG pelvis did not reveal the device. CT of the abdomen and pelvis was planned which revealed the copper T device partly in the rectosigmoid and partly in the pelvis [Figure 2]. She also had minimal free fluid in the pelvis. Hb%, total leucocyte counts and other investigations were within normal range. Necessary consent was obtained for colonoscopy and possible laparotomy. Colonoscopy was done under sedation with injection of midazolam and pethidine to assess the possibility of extraction of the device. Colonoscopy showed a part of the limb of T device as in Figure 3. It was decided that colonoscopic extraction would be attempted failing which a laprotomy was planned. Prophylactically, she was started on injection of piperacillin-tazobactum and metronidazole. Using a biopsy forceps, the “T” end of the device was pulled into the rectosigmoid. The forceps was replaced with a snare and one limb of the “T” was secured and the device extracted through the anal opening. The recto sigmoid area was inspected and no bleeding found.

Postprocedure the patient was fine, transferred to the ward for observation. Her abdomen was soft. The patient was kept nil per oral for 24 hours and started on fluids, and was taking normal diet after 48 hours postprocedure. Antibiotics were continued for 3 days, with the patient passing stools normally and tolerating orally she was discharged on oral cefaclor 500 mg bid for five more days. The patient was followed up after 1 week, and lower abdomen pain had subsided and she had no more painful defecation

## DISCUSSION

IUCD is generally a safe mode of long-term contraception. Associated complications are bleeding, infection, ectopic pregnancy and uterine perforation. The latter is one of the most morbid complications. The perforation is thought to occur at the time of insertion or occur due to chronic inflammatory reaction to the copper-containing foreign object leading to gradual erosion through the uterine wall. It is probable that of the various factors responsible for uterine perforation by IUDs, the most important are consistency and flexion of the uterus, the type and rigidity of the IUDs and its inserters and the amount of force exerted at insertions with the result that the IUDs stops at certain points rather than proceeding to complete perforation.[2] A majority of the perforations occur during the time of insertion. The delayed onset of symptoms usually means migration of the device. Timing of IUCD insertion is flexible. Most important, women who were lactating at the time of IUD insertion were 10 times as likely to have had a uterine perforation as women with at least one live birth, but who were not lactating at the time of insertion.[1]

In this case, the possible mechanism of IUCD migration is gradual erosion of the uterine wall due to chronic inflammation. Patients with indwelling IUCD should be always alerted about the possibility of its migration; regular self-examination for missing thread is useful in early detection of migration.

A plain radiograph of the abdomen and pelvis is taken to rule out unnoticed expulsion of the IUCD or USG. CT scan is recommended for exact location of the device.[3] Treatment of migrated IUCD is mostly surgical, either laparoscopic or laparotomy. In partial migration of the device, when, asymptomatic the recommendation is to withdraw it. This avoids future complications like bowel perforation, bladder perforation and fistula formation. There are few reports where the migration has been into the rectosigmoid and the pelvis where the extraction of the device has been done manually per rectum and also using a sigmoidoscope or colonoscope.[4–6] The patients did not develop peritonitis after removal. It is likely that a contained pericolic abscess may have formed already due to the longstanding nature of this case thus preventing any feacal leak and resultant peritonitis.
